# QuickStats

**Published:** 2013-08-09

**Authors:** LaJeana D. Howie

**Figure:**
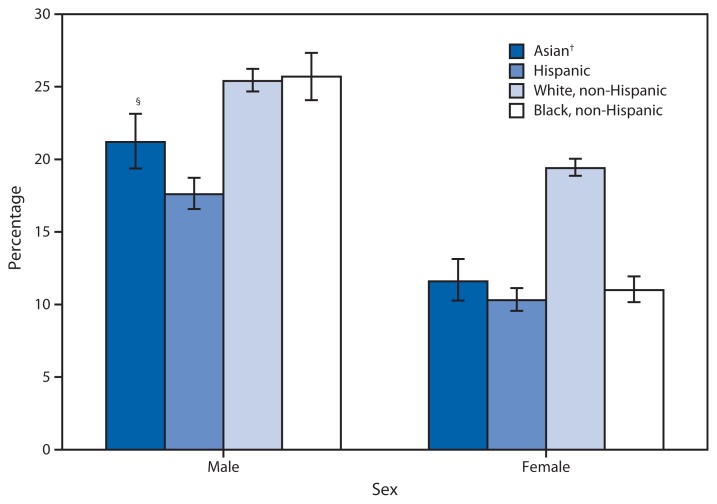
Percentage of Adults Aged ≥18 Years Who Met the Aerobic Activity and Muscle Strengthening Guidelines,^*^ by Sex and Selected Race/Ethnicity — National Health Interview Survey, United States, 2009–2011 ^*^ Respondents defined as meeting both aerobic and strengthening guidelines reported moderate physical activity for ≥150 minutes per week, vigorous physical activity for ≥75 minutes per week, or an equivalent combination of moderate and vigorous-intensity activity, and engaging in physical activities specifically designed to strengthen muscles at least twice per week. ^†^ The Asian race group includes persons who identify themselves as members of one or more Asian race subgroups. ^§^ 95% confidence interval.

During 2009–2011, males were more likely than females to have met both the aerobic activity and muscle strengthening guidelines, regardless of race/ethnicity. Non-Hispanic white and non-Hispanic black males were more likely to have met the aerobic activity and muscle strengthening guidelines compared with Hispanic and Asian males. Non-Hispanic white females were more likely to have met both guidelines compared with non-Hispanic black, Hispanic, and Asian females.

**Sources:** CDC. National Health Interview Survey, 2009–2011.

CDC. Health Data Interactive. Available at http://www.cdc.gov/nchs/hdi.htm.

